# Completion of the 1-Hour Sepsis Bundle on 90-Day Mortality in Adult Patients With Sepsis

**DOI:** 10.1016/j.acepjo.2025.100296

**Published:** 2025-12-05

**Authors:** Suraphan Charoentanyarak, Thanapong Chopetgool, Watchara Boonsawat, Kittisak Sawanyawisuth

**Affiliations:** 1 Department of Medicine, Khon Kaen Hospital, Khon Kaen, Thailand; 2 Department of Medicine, Faculty of Medicine, Khon Kaen University, Khon Kaen, Thailand

**Keywords:** age, sex, liver disease, body temperature, pulse rate, mean arterial pressure, oxygen saturation

## Abstract

**Objectives:**

Sepsis is a serious medical condition whose outcome might be improved by implementing the sepsis campaign’s 1-hour bundle. To date, there is limited evidence that completion of the bundle improves 90-day mortality in adult patients with sepsis. This study aimed to evaluate whether completion of the 1-hour sepsis bundle was effective in reducing mortality at 90 days in adult patients with sepsis.

**Methods:**

This was a retrospective cohort study that enrolled adult emergency department patients aged 18 years or older who met the criteria for sepsis or septic shock. Clinical factors were evaluated in eligible patients, who were then categorized into 2 groups: survivors and nonsurvivors. Factors predictive of mortality were calculated using logistic regression analysis.

**Results:**

During the study period, 1617 patients met the study criteria. Of these, 605 patients (37.14%) died within 90 days. The predictive model for 90-day mortality included 6 factors. Of these, 5 factors were significantly associated with mortality: age, male sex, advanced liver disease, oxygen saturation, and completion of the 1-hour sepsis bundle. Completion of the 1-hour sepsis bundle demonstrated a decreased likelihood of death with an adjusted odds ratio of 0.75 (95% CI, 0.60-0.92).

**Conclusion:**

Completion of the 1-hour sepsis bundle may lower 90-day mortality in patients with sepsis. Additionally, age, male sex, advanced liver disease, and oxygen saturation were other clinical factors predictive of mortality in this setting. Further studies are required to confirm the results of this study, particularly in other hospital settings.

**The Bottom Line**

Sepsis is associated with serious morbidity and mortality. In this study of 1617 patients with sepsis, completion of the 1-hour sepsis bundle was associated with a 25% lower mortality at 90 days. Completion of the 1-hour sepsis bundle may decrease mortality in patients with sepsis.

## 1 Introduction

### 1.1 Background

Sepsis is a life-threatening condition involving organ dysfunction that can lead to septic shock, morbidity, and mortality.^1^ A global burden of disease report stated that 48.9 million cases of sepsis occur worldwide and cause 11 million deaths.^2^ A systematic review in Europe, North America, and Australia also reported that the 90-day mortality rate in patients with sepsis was 32.2%.^3^ Moreover, the mean hospital cost for patients with sepsis may be as high as €91,951 per patient per admission.^4^

The early treatment of sepsis was introduced by the Surviving Sepsis Campaign (SSC) in 2004 with the aim of improving the quality of care for the condition.^5^ In 2018, the SSC introduced the 1-hour sepsis bundle, which comprises 5 components: measuring lactate level, obtaining blood cultures prior to antibiotic administration, administering broad-spectrum antibiotics, administering intravenous fluids, and applying vasopressors to keep mean arterial pressure (MAP) at 65 mm Hg or more.^5^ The 1-hour sepsis bundle has since been implemented worldwide.

Several studies have shown that the 1-hour sepsis bundle is effective in reducing mortality in patients with sepsis.^6^, ^7^, ^8^, ^9^ For example, a cohort study in New York revealed that completion of the 1-hour sepsis bundle lowered in-hospital mortality (odds ratio, 0.59) in pediatric patients with sepsis.^8^ However, other studies found no improvement in mortality in various settings, including for in-hospital and 28-day and 30-day mortality.^10^, ^11^, ^12^, ^13^, ^14^, ^15^ One study of adult patients with sepsis in emergency departments (EDs) found an insignificant reduction in 28-day mortality in patients who completed the 1-hour sepsis bundle vs those who had not.^10^ Given the limited evidence regarding the effect of the 1-hour sepsis bundle on 90-day mortality, we aimed to evaluate the bundle’s effectiveness for that outcome. Predictors of the 90-day mortality rate in this setting were also investigated.

### 1.2 Importance

This study may show that completion of the 1-hour sepsis bundle has long-term mortality benefits for patients with sepsis. Increased physician implementation of the 1-hour sepsis bundle in the ED may lower long-term mortality rates in this setting.

### 1.3 Goal of This Investigation

To evaluate whether completion of the 1-hour sepsis bundle can lower the 90-day mortality rate. Additionally, predictors of the 90-day mortality rate are also investigated.

## 2 Methods

### 2.1 Study Design

This was a retrospective cohort study.

### 2.2 Setting

The Emergency Department of Khon Kaen Hospital, a tertiary care facility in Thailand.

### 2.3 Selection of Participants

The inclusion criteria were adult ED patients aged 18 years or older who met the criteria for sepsis or septic shock. Both sepsis and septic shock were defined according to the SSC’s 2016 International Guidelines for Management of Sepsis and Septic Shock.^1^ In the study, conducted from January 1, 2020, to February 28, 2021, the exclusion criteria were being pregnant, having end-stage disease, or being referred from other hospitals. The study protocol was approved by Khon Kaen Hospital’s Institutional Review Board for Human Research (KE63053), and the STrengthening the Reporting of OBservational studies in Epidemiology (STROBE) guidelines for cohort studies were used for reporting.^2^ Study variables with >50% missing data were excluded from our analysis.^3^

### 2.4 Measurements

Eligible patients were identified from a hospital database and followed for 90 days to determine the mortality. The studied variables included baseline characteristics, physical signs, blood culture results, treatments, and completion of the 1-hour sepsis bundle. Baseline characteristics were age, sex, and comorbidities, while physical signs included temperature, pulse rate, respiratory rate, systolic and diastolic blood pressure, MAP, shock defined as a MAP <65 mm Hg, level of consciousness (“alert,” “drowsy,” “unresponsive,” or “coma”), and oxygen saturation. Blood culture results were reported along with therapeutic interventions, including fluids, duration of renal replacement therapy (in days), duration of endotracheal intubation (in days), and completion of the 1-hour sepsis bundle. Completion of the 1-hour sepsis bundle was defined as the completion of the 5 components mentioned in the introduction.^5^

### 2.5 Outcomes

The primary outcome of the study was 90-day mortality, recorded by using the national database of population. In-hospital mortality and length of stay were also recorded. All therapeutic interventions were provided by the attending physicians according to each patient’s clinical condition.

### 2.6 Data Analyses

Patients were categorized into 2 groups, survivors and nonsurvivors, based on their 90-day outcome. Variables, including baseline characteristics, physical signs, blood culture results, treatments, and outcomes, were compared. Categorical variables were recorded as counts (*n*) and percentages (%), whereas continuous variables were recorded as mean ± SD for normally distributed data or as median (IQR) for nonnormally distributed data. Inferential statistics were used to compare differences between the groups, including the Student *t* test or Wilcoxon rank-sum test for continuous variables and the chi-squared test or Fisher exact test for categorical variables. The comparative analyses were subsequently repeated with patients stratified according to their completion of the 1-hour sepsis bundle.

Factors predictive of 90-day mortality were identified using logistic regression analysis. Variables with a *P* value of <.05 in the univariable analysis were included in the subsequent multivariable logistic regression model. Both unadjusted and adjusted odds ratios (aOR) with their 95% CIs were reported for the predictive model. The Hosmer-Lemeshow test was used to evaluate the model’s goodness of fit. All statistical analyses were performed in Stata version 18.0 (StataCorp LLC).

## 3 Results

Of the 1617 patients who met the inclusion criteria, 605 (37.4%) died within 90 days of admission. Among the baseline characteristics, 7 factors differed significantly between the survivor and nonsurvivor groups including the following: (1) sex, (2) diabetes mellitus, (3) advanced liver disease, (4) pulse rate, (5) respiratory rate, (6) oxygen saturation, and (7) Sequential Organ Failure Assessment (SOFA) score (Table 1). Compared with survivors, nonsurvivors were more likely to be men (70.6% vs 64.3%), diabetic (29.6% vs 24.6%), and had a higher prevalence of advanced liver disease (14.1% vs 7.2%). Nonsurvivors had a higher pulse rate (112.1 vs 108.7 beats/min), respiratory rate (29.4 vs 27.9 breaths/min), and median SOFA score (5.3 vs 3.2) but a lower mean oxygen saturation (92.1% vs 95.1%). Significant differences between the groups also emerged regarding treatments, including fluid therapy, duration of renal replacement therapy, and duration of endotracheal intubation. Nonsurvivors had a significantly lower rate of 1-hour sepsis bundle completion than survivors (55.0% vs 61.7%). Among nonsurvivors, 529 patients (87.4%) died during their hospital stay (ie, in-hospital mortality). Survivors had a longer median hospital length of stay than nonsurvivors (9.4 vs 7.2 days).

**Table 1:** Baseline characteristics, physical signs, laboratory results, and management of patients with sepsis categorized by survival outcomes (n = 1617).

Factors	Survivors n = 1012	Nonsurvivors n = 605	Mean difference/odds ratio	95% CI
Baseline characteristics				
Age, y	62.4 (16.0)	63.7 (14.9)	−1.3	−2.9 to0.2
Male sex	651 (64.3)	427 (70.6)	1.33	1.07-1.65
Comorbid diseases	740 (73.1)	458 (75.7)	1.15	0.91-1.44
Diabetes mellitus	300 (29.6)	149 (24.6)	0.71	0.53-0.95
Advanced liver disease	73 (7.2)	85 (14.1)	2.11	1.47-3.01
CKD 3-5	120 (11.9)	69 (11.4)	0.96	0.70-1.31
Physical signs				
Body temperature, °C	38.2 (1.3)	37.8 (1.3)	0.4	0.2-0.5
Pulse rate, bpm	108.7 (23.4)	112.1 (24.6)	−3.4	−5.8 to −0.9
Respiratory rate, tpm	27.9 (9.9)	29.4 (8.2)	−1.5	−2.4 to −0.6
Mean arterial pressure, mm Hg	87.1 (23.2)	82.4 (22.2)	4.7	2.4-7.0
Consciousness				
Alert	865 (85.5)	423 (69.9)	1	Reference
Drowsiness	83 (8.2)	82 (13.6)	2.02	1.46 to 2.80
Stuporous	31 (3.1)	42 (6.9)	2.77	1.72-4.47
Coma	33 (3.3)	58 (9.6)	3.59	2.31-5.60
O_2_ saturation, %	95.1 (6.5)	92.1 (9.2)	3.0	2.3-3.8
SOFA	3.2 (2.8)	5.3 (5.6)	−2.1	−2.4 to −1.7
Hemoculture				
No growth	866 (85.7)	489 (81.0)	1	Reference
Gram positive	32 (3.2)	39 (6.5)	2.16	1.33-3.49
Gram negative	113 (11.2)	76 (12.6)	1.19	0.87-1.63
Fluid therapy				
Normal saline	821 (81.1)	521 (86.1)	1.90	0.20-18.35
Ringer lactate	54 (5.3)	17 (2.8)	0.94	0.09-9.68
Acetar	134 (13.2)	66 (10.9)	1.48	0.15-14.48
Treatment				
Renal replacement therapy, d	0.3 (1.8)	0.8 (3.8)	−0.5	−0.8 to −0.2
Endotracheal tube, d	2.0 (4.5)	4.6 (6.4)	−2.6	−3.2 to −2.1
Duration of inotropic treatment, d	0.7 (1.4)	1.9 (2.5)	−1.1	−1.3 to −1.0
Completion of the 1h bundle	624 (61.7)	333 (55.0)	0.76	0.62-0.93
Outcomes				
In-hospital death	0	529 (87.4)	NA	
Length of hospital stay, d	9.4 (9.9)	7.2 (8.1)	2.3	4.3-3.2

CKD, chronic kidney disease; NA, not available.

Data presented as median (IQR) or number (percentage) unless indicated otherwise.

Table 2 shows the differences between patients who received the complete 1-hour sepsis bundle and ones who did not. Five factors differed significantly between the groups: sex, body temperature, respiratory rate, MAP, and SOFA score. The group that completed bundle implementation had a higher proportion of male patients, higher body temperature, higher respiratory rate, higher MAP, and a higher SOFA score than the group that did not complete bundle implementation.

**Table 2:** Baseline characteristics, physical signs, laboratory results, and management of patients with sepsis categorized by completion of the 1-hour sepsis bundle (n = 1617).

Factors	Incomplete n = 660	Complete n = 957	Mean difference/odds ratio	95% CI
Baseline characteristics				
Age, y	63.6 (14.8)	62.6 (16.2)	0.7	−0.9 to2.2
Male sex	419 (63.5)	659 (68.9)	1.27	1.03-1.57
Comorbid diseases	480 (72.7)	718 (75.0)	1.13	0.90-1.41
Diabetes mellitus	184 (27.9)	265 (27.7)	1.18	0.89-1.57
Advanced liver disease	74 (11.2)	84 (8.8)	0.77	0.54-1.10
CKD 3-5	74 (11.2)	115 (12.0)	1.08	0.79-1.48
Physical signs				
Body temperature, °C	37.9 (1.3)	38.1 (1.4)	−0.2	−0.3 to −0.1
Pulse rate, bpm	108.9 (24.0)	110.8 (23.8)	−1.9	−4.2 to 0.5
Respiratory rate, tpm	27.5 (9.4)	29.1 (9.3)	−1.6	−2.5 to −0.7
Mean arterial pressure, mm Hg	82.7 (22.5)	87.2 (23.1)	−4.5	−6.8 to −2.2
Consciousness				
Alert	512 (77.6)	776 (81.1)	1	Reference
Drowsiness	76 (11.5)	89 (9.3)	0.77	0.56-1.07
Stuporous	27 (4.1)	46 (4.8)	1.12	0.69-1.83
Coma	45 (6.8)	46 (4.8)	0.67	0.44-1.03
O_2_ saturation, %	94.3 (7.3)	93.7 (8.1)	0.6	−1.2-1.4
SOFA	4.4 (3.5)	3.6 (3.0)	0.8	0.5-1.1
Hemoculture				
No growth	549 (83.4)	806 (84.2)	1	Reference
Gram positive	34 (5.2)	37 (3.9)	0.74	0.46-1.20
Gram negative	75 (11.4)	114 (11.9)	1.04	0.76-1.41
Fluid therapy				
Normal saline	549 (83.4)	783 (81.8)	1	Reference
Ringer lactate	23 (3.5)	48 (5.0)	1.49	0.90-2.48
Acetar	74 (11.4)	126 (13.2)	1.22	0.89-1.65
Treatment				
Renal replacement therapy, d	0.5 (2.5)	0.5 (2.9)	0.1	−0.2 to 0.3
Endotracheal tube, d	3.3 (5.9)	2.7 (5.1)	0.7	0.1-1.2
Duration of inotropic treatment, d	1.4 (2.1)	1.0 (1.8)	0.4	0.2-0.6
Outcomes				
Death at 90d	272 (41.2)	333 (34.8)	0.76	0.62-0.93
In-hospital death	242 (36.7)	287 (30.0)	0.74	0.60-0.91
Length of hospital stay, d	9.1 (10.0)	8.3 (8.8)	0.8	−0.1 to 1.7

CKD, chronic kidney disease; NA, not available.

Data presented as median (interquartile range) or number (percentage) unless indicated otherwise.

The final predictive model for 90-day mortality included 6 factors, 5 of which were significantly associated with mortality: age, male sex, advanced liver disease, oxygen saturation, and completion of the 1-hour sepsis bundle (Table 3). Completion of the 1-hour sepsis bundle was associated with a protective effect, with an aOR of 0.75 (95% CI, 0.60-0.92). The Hosmer-Lemeshow test indicated good model calibration, with a chi-squared value of 4.48 (*P =* .812).

**Table 3:** Factors associated with mortality at 90 days in patients with sepsis by logistic regression analysis.

Factors	Unadjusted odds ratio (95% CI)	Adjusted odds ratio (95% CI)
Age	1.00 (0.99-1.01)	1.01 (1.00-1.02)^a^
Male sex	1.33 (1.07-1.65)	1.34 (1.06-1.68)
Diabetes mellitus	0.71 (0.53-0.95)	0.77 (0.57-1.04)
Advanced liver disease	2.11 (1.47-3.01)	2.23 (1.24-3.23)
Oxygen saturation	0.95 (0.94-0.96)	0.95 (0.94-0.96)
Completion of the 1-h sepsis bundle	0.76 (0.62-0.93)	0.75 (0.60-0.92)

a *P*< .05.

## 4 Strengths and Limitations

A key strength of our study was its identification of baseline clinical factors and physical signs that may help physicians to predict 90-day mortality from sepsis. Moreover, our findings support the importance of completion of the 1-hour sepsis bundle. Even so, our findings have some limitations. First, the infections studied were exclusively community acquired. Second, treatments were administered at the discretion of the attending physicians without a standardized protocol. Third, laboratory data and specific pathogens were not included in the predictive model.^4^^,^^5^ Details regarding the timing and specific components of the 1-hour sepsis bundle were not analyzed, and completion was recorded as complete or incomplete^6^^,^^7^; as a consequence, it is possible that unknown confounders, including delays caused by urgent airway management, influenced bundle completion. The relatively small difference in the rates of bundle completion between survivors and nonsurvivors may reflect the inherent inability of retrospective studies to control for such confounders. Lastly, clinical endpoints such as quality of life and cost-effectiveness were not evaluated.^8^ Despite these limitations, completion of the 1-hour sepsis bundle was associated with a 25% lower odds of mortality (Table 3), which is a finding with clear clinical importance. The high overall mortality rate observed in our cohort may be attributable to the heterogeneity of treatments. Further prospective studies are warranted to confirm those findings and address the mentioned limitations.

## 5 Discussion

Completion rate of the 1-hour sepsis bundle in our study was 37.14%, a rate that was somewhat higher than in a study on adults in South Korea (28.6%) and in the New York Sepsis Care study with pediatric patients (24.9%).^9^^,^^10^ However, the completion rate in our study was lower than that reported by the SSC (62%).^11^ Similar to our study, the studies conducted in South Korea and New York may reflect real-world practice. In our study’s setting, a sepsis protocol was implemented and introduced to emergency physicians, which likely contributed to the slightly higher completion rate observed.

Completion of the 1-hour sepsis bundle significantly lowered the 90-day mortality rate compared with incomplete bundle implementation (Tables 1 and 2). These results underscore the importance of early sepsis detection and treatment. Similarly, a study from China involving 153 adult patients with sepsis showed that the 28-day survival rate was significantly higher in the group that received the complete 1-hour bundle than in the group that did not (80.00% vs 62.06%; *P =* .014).^12^ Extending that finding, our study involved evaluating mortality over a longer term, at 90 days. Our predictive model likewise differs from models in other studies that did not show a significant association with bundle completion.^7^^,^^10^^,^^13^, ^14^, ^15^ The discrepancy may stem from our longer timeframe of evaluation.

Among other notable results, patients who received the complete 1-hour bundle tended to be men and presented with more severe clinical features, including higher body temperature and higher respiratory rate (Table 2). Those outcomes suggest that attending physicians prioritized bundle completion for patients who appeared more critically ill, even if their MAP was not yet low.

We also found that age, being men, advanced liver disease, and oxygen saturation were significantly associated with 90-day mortality in patients with sepsis (Table 3). As previously reported, older age was a significant predictor of mortality.^6^ The aOR for age in our study (1.01) was slightly lower than that in a previous study conducted in patients aged >80 years (aOR = 1.019). Even though those who received the complete 1-hour bundle were more men (68.9% vs 63.5%), male sex was a significant factor associated with mortality at 90 days. These results were compatible with previous studies showed that male patients with sepsis may have a higher mortality rate, particularly at 1 year.^16^^,^^17^ Regarding comorbidities, only advanced liver disease was independently associated with 90-day mortality. The result is consistent with the findings of past research showing that patients with cirrhosis have an exceptionally high mortality rate from sepsis; in several studies, >60% of patients with cirrhosis were found to die within 30 days and to have a fourfold increased risk of mortality.^18^, ^19^, ^20^ Although diabetes mellitus was significant in the univariable analysis, it lost its significance in the multivariable model (Table 3). That result suggests that the effect of diabetes mellitus was not strong enough to remain an independent predictor when considered alongside other factors, particularly advanced liver disease.

Finally, a low oxygen saturation at presentation was also associated with mortality, which is consistent with its known association with severe organ dysfunction, particularly lung injury, in sepsis.^21^ In patients with pneumonia, an oxygen saturation <90% has been shown to correlate with higher mortality (6% vs 1%; *P* < .001). Our data suggest that a threshold of <95% may be associated with increased mortality as well (Table 1).

Altogether, completion of the 1-hour sepsis bundle may lower 90-day mortality in patients with sepsis. Age, being men, advanced liver disease, and oxygen saturation were clinical factors predictive of mortality in the population studied. To confirm our results, additional studies are required, particularly in other hospital settings.

## Author Contributions

SC and TC conceived the study, designed the protocol, and collected data. WB supervised the study. KS analyzed the data. SC drafted the manuscript, and all authors contributed substantially to its revision. SC takes responsibility for the paper in its entirety.

## Funding and Support

By *JACEP Open* policy, all authors are required to disclose any and all commercial, financial, and other relationships in any way related to the subject of this article as per ICMJE conflict of interest guidelines (see www.icmje.org). The authors have stated that no such relationships exist.

## ORCID

Kittisak Sawanyawisuth, MD, PhD https://orcid.org/0000-0003-3570-8474

## Conflict of Interest

All authors have affirmed they have no conflicts of interest to declare.
